# Supplementary material for: Leishmanicidal and healing effects of 3β,6β,16β-trihydroxy lup-20 (29)-ene isolated from Combretum leprosum on Leishmania braziliensis infection in vitro and in vivo
Source: PLoS One. 2023 Nov 27;18(11):e0287665. doi: 10.1371/journal.pone.0287665 (PMC10681296; doi:10.1371/journal.pone.0287665)
Supplement: Attachment — Submitted filename: ResponsetoReviewers.docx [file pone.0287665.s001.docx]

May 8th, 2023

The Editor

PLOS ONE

Dear Editor,

We are respectfully resubmitting a revised version of the manuscript “Leishmanicidal and healing effects of 3β,6β,16β-trihydroxy lup-20 (29)-ene isolated from *Combretum leprosum* on *Leishmania braziliensis* infection *in vitro* and *in vivo*” for consideration for publication in PLOS ONE.

We are appreciative of the reviewers’ comments and have carefully addressed all the points raised. To facilitate the review, our answers are written in italics and the changes made to the manuscript are underlined here and highlighted the revised manuscript.

We would like to include another author, Bianca Oliveira Loucard, since she contributed with the formulation of CLF-1 cream and analysis of experiment results.

ANSWERS TO REVIEWERS:

**Reviewer #1:** In this article by Santos et al, entitled Leishmanicidal and healing……… on *Leishmania braziliensis* infection in vitro and in vivo” the authors have demonstrated anti-leishmanial and wound healing effect of *Combretum leprosum* leaves extract. The article has important information and findings but requires revision with clarification before being accepted for publication.
My comments are below.

1. Brief description of isolation and characterization of CLF-1 in page 5 line 106 should be provided.

*More information related to the isolation and characterization of CLF-1 was included in the Material and Methods section as suggested.*

2. The cream formulation should be elaborated in page 6 line 135.

*A more detailed description of CLF-1 cream formulation was included in the Material and Methods section as suggested.*

3. In figure 1, panel A, B, C & D, the only macrophage bar in all the cases- it is not clear against which control was these bars normalized. In panel ‘A’ the macrophage bar is around 120 %, in panel ‘B’ it is around 130%, in ‘C’ it is 110% and in ‘D’ it is less than ‘100’. Please clarify and correct. It is also unclear why 100µg/ml CFL-1 is toxic to macrophage but not to Leishmania in that respect.

*We appreciate that the reviewer’s comment and we have corrected figure 1, the bars were normalized based on the experiment controls. While CLF-1 cytotoxicity to macrophages was only observed with the highest concentration (100µg/ml), promastigotes were more susceptible to the toxic effect, observed also with lower CLF-1 concentrations (10 µg/ml). This is probably resulting from different mechanisms of actions of CLF-1 on such distinct cells (macrophages and Leishmania parasites) and should be further explored on future studies.*

4. In figure 2 the authors should explain why there is not much effect of duration of treatment.

*We believe that we did not observe a significant effect on amastigotes at 24h, but not at 48h, probably due to the kinetics of CLF-1 effect on the intracellular parasites. Unfortunately, we were not able to test in vitro for a longer period. However, the CLF-1 anti-leishmanial effect was evident in vivo on infected hamsters where we observe a clear reduction of parasite numbers and lesions were not ulcerated with a necrotic center compared to the group treated with Glucantime.*

5. The data provided in the manuscript shows that higher dose of CFL-1 has no effect on number of amastigotes (Fig 2) but higher dose of CFL-1 do induce the cytokines (Fig 3). How can this be explained in wound healing and clearance of parasites.

*The mechanism of CLF-1 treatment on amastigotes (intracellular) and macrophages and the kinetics of the effect observed is probably different and it remains to be elucidated. CLF-1 has been previously described as having an antimicrobial, pro-healing and antiparasitic activities. The wound healing and clearance of parasites is probably resulting, at least in part, by direct modulation of cytokine production that results in parasite killing and promote lesion healing.*

6. I would suggest the authors to measure the parasitic load through some molecular method like qPCR.

*Although qPCR is a sensitive and specific method, limiting dilution is more accessible, widely used, and a reliable method to quantify live parasites. Thus, we decided to measure the parasite burden using the limiting dilution method to determine the parasite load. This method has been used by our group in other studies (Dutra, et al., 2020 and Freire et al., 2022).*

**Reviewer #2:** Author has shown with different experiments that CLF-1 has *in vitro* (in J774 macrophage) and *in vivo* (in Hamster model) anti-leishmanial effect against *L. braziliensis*. However, author should address the following comments before acceptance.

Comments:

1. Author did not study any mechanism of inhibition for CLF-1 in *L. braziliensis* as previous study already explored the mechanism of inhibition by CLF-1 in *L. amazonensis* promastigotes and also showed that CLF-1 has anti-leishmanial activity in-vitro. So, in my opinion the only novel part of this study was to explore the effect of CLF-1 cream in Hamster model. Author should study the mechanism of inhibition in amastigote by isolating the amastigotes from infected macrophages.

*The reviewer made an important point about previous work demonstrating the potential protective and healing effect of CLF-1. Infection caused by Leishmania braziliensis and Leishmania amazonensis induce skin lesions, but disease progression and immune evasion mechanisms are distinct reinforcing the importance of testing CLF-1 against each individual Leishmania species. Investigation of the mechanism of action of CLF-1 treatment on amastigotes and macrophages are the subject of a future study.*

2. Author used J774 (mouse cancerous cell line) for in-vitro infection study. In my opinion, author may use mouse peritoneal macrophages for this study as this the primary macrophages.

*Although we have not used a primary culture of macrophages due to a limitation on the number of experimental hamsters/mice available at the time, we believe that J774 cells, that are widely used for in vitro tests, also provide reliable and representative results.*

3. For in-vitro study, author used Amphotericin B as a control and concentration used is 16 µg/ml. What is the rationale behind choosing this concentration? Is there any report?

*We have used this concentration of Amphotericin B as a control for parasite killing. It was based on previous work published by the group (Pinheiro et al., 2021). This reference was included in the Material and Methods.*

4. Author has developed CLF-1 cream for topical application. However, author did not provide the details about the process of formulation of the cream.

*Details concerning the formulation of CLF-1 cream were included in the Material and Methods section as suggested.*

5. Author has shown that promastigotes viability is reduced with increasing concentration of CLF-1 (Figure 1) and in case of intracellular amastigotes, viability is increased with increasing concentration of CLF-1 (Figure 2). What is the explanation of this contradictory results? However, I am quite happy with the work but, author need to address the above issues.

*We observed a different effect on promastigotes and amastigotes probably as a result of the mechanism of action and kinetics of CLF-1 on the different stages (intracellular x extracellular). Unfortunately, we were not able to test in vitro for a longer period as we moved forward for in vivo experiments. The CLF-1 anti-leishmanial effect was evident in vivo on infected hamsters where we observe a clear reduction of parasite and lesions were not ulcerated with a necrotic center compared to the group treated with Glucantime.*
